# Next-Generation Approaches Needed to Tackle Antimicrobial Resistance for the Development of Novel Therapies Against the Deadly Pathogens

**DOI:** 10.3389/fphar.2022.838092

**Published:** 2022-06-02

**Authors:** Vasundhra Bhandari, Akash Suresh

**Affiliations:** ^1^ National Institute of Pharmaceutical Education and Research (NIPER)– Hyderabad, Hyderabad, India; ^2^ National Institute of Animal Biotechnology, Hyderabad, India; ^3^ Manipal Academy of Higher Education (MAHE), Hyderabad, India

**Keywords:** antimicrobial resistance, antivirulent drugs, antimicrobial peptides, extended spectrum drug resistance, phage therapy

## Abstract

The advent of antimicrobials was a miracle that saved millions of lives across the globe. With the discovery of penicillin, varieties of other antimicrobials came into play one after another. However, the injudicious use of antimicrobials for therapeutics and prophylactics and overuse in agriculture and animal husbandry industries resulted in its gloominess and rise of antimicrobial resistance. The microbes have slowly outsmarted the human race with diverse mechanisms to evade the antimicrobial effects of the drugs in use. The review aims to discuss the emergence of resistance in bacterial species with time and the various means by which bacterial cells had safeguarded themselves. In addition to that, we have also highlighted new approaches currently used to tackle antimicrobial resistance or practices that could be useful in identifying new treatment options.

## Introduction

During the pre-antibiotic era, high morbidity and mortality were observed due to minor infections. Then, thanks to the great minds of Sir Alexander Fleming and Paul Ehrlich, antimicrobials were discovered and helped humanity sustain the fatal blow from microbial infections (Gaynes, 2017). Nevertheless, the return of those days is not much far away. The World Health Organization (WHO) has already predicted a surge of 1.2 trillion USD in annual health expenditure by 2050, sinking the global GDP by 1.1–3.8%, because of antimicrobial-resistant pathogens (CDC, 2020). A 2 year review commissioned by the United Kingdom Government concluded in 2016 has shown that annually 700,000 deaths happen due to AMR infections (Woolhouse and Farrar, 2014).

**FIGURE 1:**
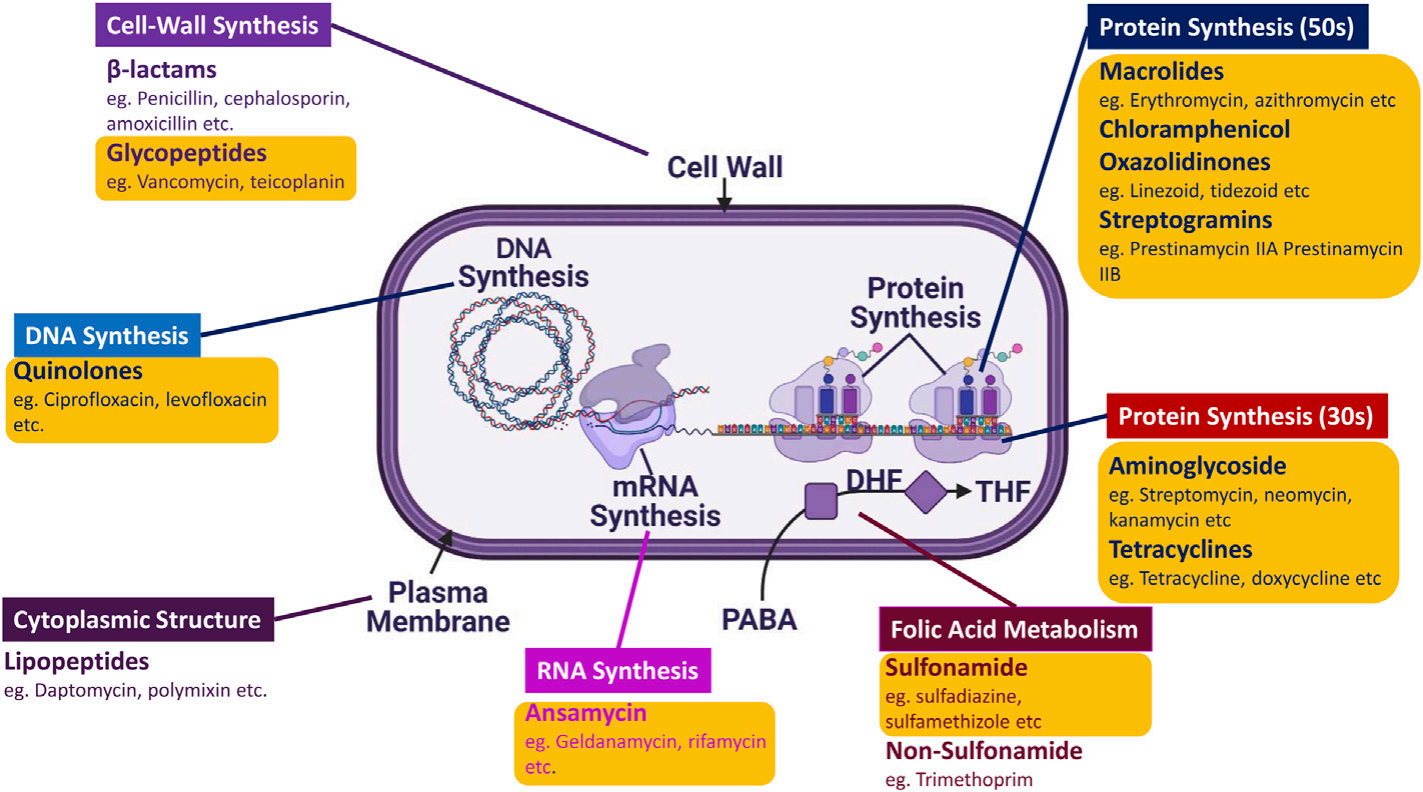
Schematic representation of AMDs distribution according to their targets: There are 13 significant classes of AMDs that target different types of machinery and aspects of the microbe, ranging from DNA synthesis to cell wall synthesis. The majority of the AMDs marked in yellow (O) box belong to the Golden Era.

The timeframe from 1950 to the late 1970s marked the Golden era of antibiotics due to the significant influx of AMDs (Figure 1) caused by the potential success rate and economic benefits insight of pharmaceutical companies. The majority of all the antibiotics classes were found during this period, encompassing approximately 70 drugs of varying categories. These classes were divided based on targets and chemical structures (Firth and Skurray, 1998). However, Sir Fleming had warned us of antimicrobial resistance; all it took was 5 years for the emergence of Penicillin-resistant *S. aureus* in 1947 post the Penicillin mass production in 1942 (Waksman, 1947). Somehow, this was not a primary concern back then due to the constant inflow of new drugs by the day.

**FIGURE 2:**
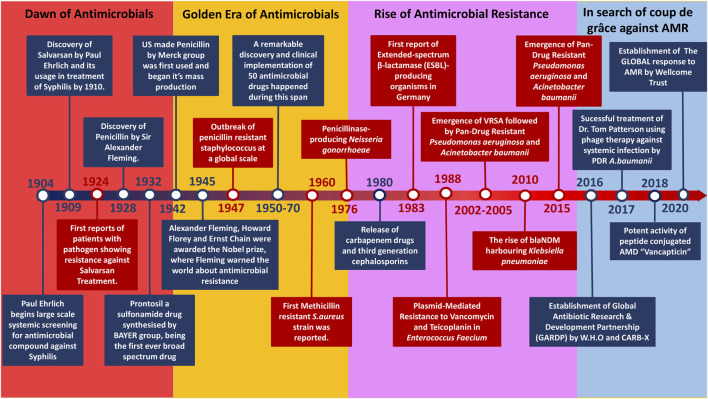
Timeline of events leading to the rise and fall of AMDs across different eras.

Antimicrobial compounds produced were by microbes to outcompete rivalry or defence mechanisms against microbes (Davies and Davies, 2010)^,^ (Beckh and Kulchar, 1939). The evolution of the resistance mechanisms helped the other microbes to withstand the stress (Figure 2). The rise of first-ever antimicrobial-resistant bacteria is suspected to be through random gene transfer events initially by bacteriophage-driven transduction (Magiorakos et al., 2012). However, the drastic usage of AMDs led to the Darwinian selection of the resistant bacteria and causing their rise among the population. The horizontal gene transfer among bacterial species caused further spread of antimicrobial genes resulting in antimicrobial-resistant phenotype (CDC, 2021). In 1924 the first-ever case of antimicrobial resistance was reported, which dates even before the discovery of Penicillin. The resistance reported was against a compound called Salvarsan, an Arsphenamine discovered by Paul Ehrlich in 1910 used to treat syphilis until early 1940 (Walsh, 2003). Parallel to the emergence of newer AMDs of varying classes, microbes also responded with different antimicrobial resistance mechanisms. Humanity thrived on various combination therapies and synthetic drugs as a counter to antimicrobial-resistant pathogen infection. Currently, various common pathogens have found resistance even against their last-resort respective medicines (CDC, 2020). The constant misuse and improper prescription of these drugs have fastened the course of AMR pathogen domination. Unlike their predecessor, these Multidrug-Resistant (MDR) bacteria are non-susceptible to at least one agent in three or more antimicrobial categories (Werner et al., 2008). This broad spectrum of antimicrobial resistance started to widen further, resulting in the rise of Extended-Spectrum Drug Resistance (XDR) and later on the increase of Pandrug Resistance (PDR) (Werner et al., 2008). Things took a turn once antimicrobial resistance was on the rise with the downfall of AMD discovery. Hence, we want to shed light on the current scenario and future in the battle against AMR bacteria through this review.

## Next Generation Approach to Tackle Antimicrobial Resistance

**FIGURE 3:**
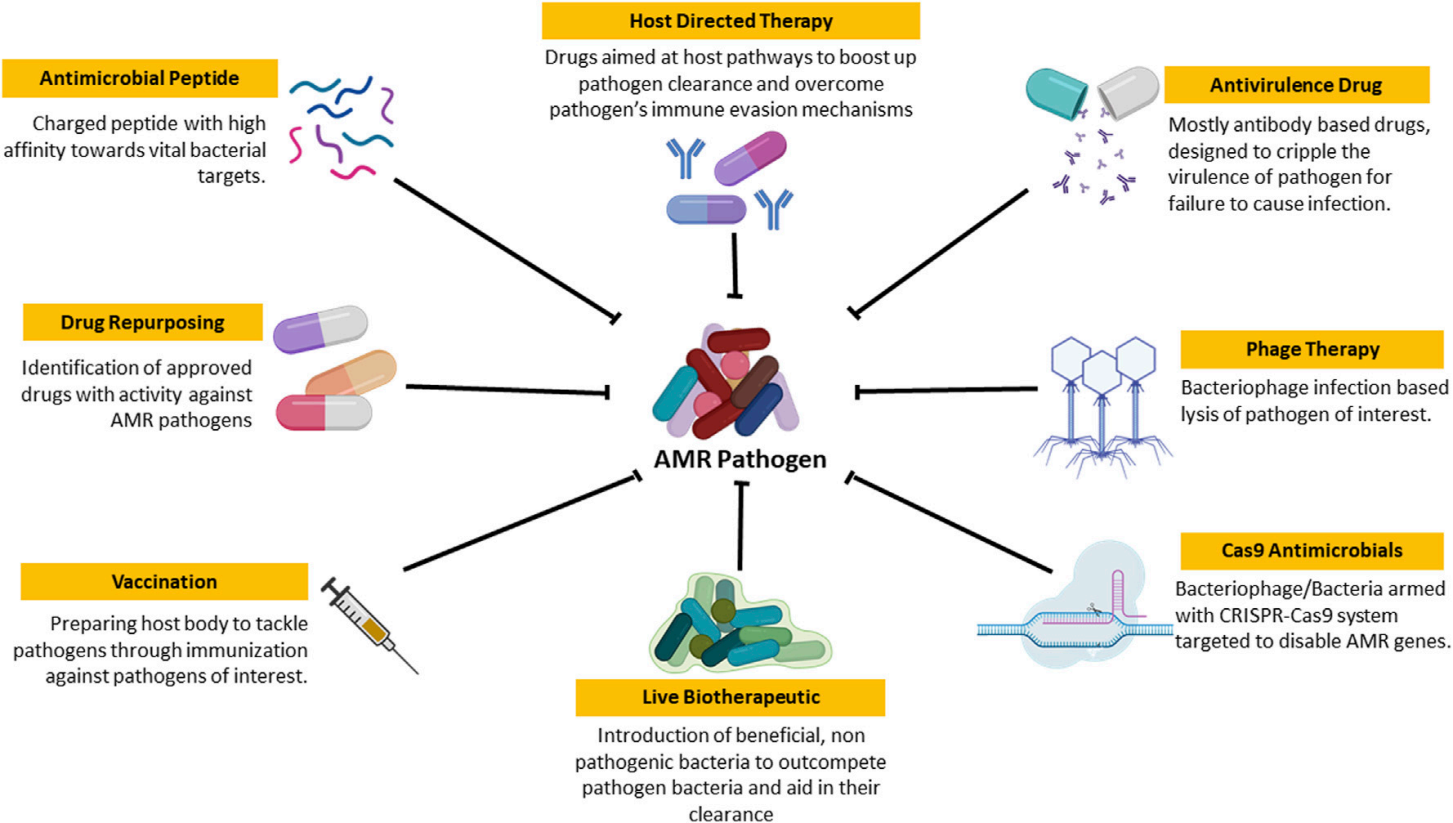
Schematic representation of newer approaches to tackle AMR Pathogen: These are the set of approaches that are currently under research for efficient treatment of AMR pathogen based infection and its spread.

As the clock ticks, the downfall of conventional AMDs is becoming more and more evident. Hence, there is a requirement for a newer, efficient, and durable approach to tackle the situation. Currently, many people are working on various aspects to tackle AMRs ranging from antimicrobial peptides to phage therapy as shown in Figure 3.

### Antivirulence Drugs

AMD has been targeting the essential components of the pathogens, which has caused the pathogens to go under selection pressure and evolve at a tremendous rate. So the next step to be taken is to target the non-essential aspects of the pathogen to avoid such selection (Cegelski et al., 2008)^,^ (Dickey et al., 2017). This very low probability of resistance development is why AVDs are currently trending mechanisms to treat pathogenic infections. They intend to target the virulence factors of pathogens, crippling their efficiency to cause disease. This disarming shall help eradicate the pathogen in a much healthier way (Arnon et al., 2006). The host’s microflora is not damaged, and selection pressure gets minimized to avoid the evolution of resistance against the therapy. Currently, there are a couple of AVDs that got approved by the FDA, which are primarily Monoclonal Antibody (MAb) based or compound based inhibitors (Migone et al., 2009). These AVDs have managed to improve the eradication of infectious pathogen but also in subduing the inflammatory response triggered by the toxins released by the pathogen. The BabyBIG, BAT, raxibacumab, bezlotoxumab and Obiltoxaximab are all FDA approved antitoxins that reduce the stress caused by the pathogen on the host body (Hancock and Chapple, 1999; Khardori, 2010; Greig, 2016; Mahlapuu et al., 2016). This window gives the host body enough time to act. However, the lack of antivirulence drugs to show equal effectiveness as lab tests in the real world is a significant hindrance in the path of its successful establishment as reliable form of therapy.

### Antimicrobial Peptides

AMPs typically charged peptides produced by all living organisms as a defence mode against pathogens or outcompete rival microorganisms to colonize the same niche (Huan et al., 2020). Even though there are umpteen number of ways to classify AMPs they can be broadly classified into two classes based on their natural synthesis, ribosome dependant and ribosome independent synthesis (Hancock, 2000)^,^ (Nguyen et al., 2011). In which the ribosome-dependant ones seen are in eukaryotes and ribosome-independent mechanisms in prokaryotes (Huan et al., 2020). However the non-ribosomal AMPs were known for their antimicrobial properties, the ribosomal AMP’s therapeutic potential has gained much attention in recent times. These AMPs interacts with bacterial membrane and disrupts their membrane polarity causing membrane dysfunction (Hancock, 2000). The ability of AMPs to breach the outer membrane of Gram-negative bacteria helps AMPs work efficiently against a broader range of pathogens. In addition, the multi-targeting of AMPs helps reduce the chances of resistance development (Nguyen et al., 2011). AMPs combined with known glycopeptide antibiotics have improved the drug’s efficiency, which led to the discovery of lipoglycopeptides “Vancapticins” (Blaskovich et al., 2018). Vacapticin is a combination of a peptide with Vancomycin that has resulted in a 4 to 10 fold better activity than the unconjugated variant of Vancomycin against MRSA, VISA, and MDR *Streptococcus pneumonia* (Blaskovich et al., 2018). A further understanding of such combinations of peptides and antibiotics will help better drug repurposing in the market. However, many AMPs fail to clear the FDA drug screening procedures due to their host cytotoxicity or failure to replicate lab results in the *in vivo* studies (Kosikowska and Lesner, 2016). Further research into methods and guidelines for standardization and validation of AMP’s efficiency will help in upbringing AMPs as an efficient and reliable alternate.

### Phage Therapy

Phage therapy dates back decades, even before the discovery of Penicillin. Phage therapy is currently trending in research after the rise of multidrug resistance, but knowingly or unknowingly, phages have been used to treat microbial infections for ages. One such example is the presence of *Vibro cholerae* killer phages at high titre in the Ganges, where people took a dip and got cured of cholera back in the 1890s (Adhya and Merril, 2006). Bacteriophages are viruses that specifically infect bacterial cells. However, some phages show a broad spectrum of infectivity where they can infect various bacterial species. In contrast, some tend to be specific to a particular species or strain of bacteria (Koskella and Meaden, 2013).

Over the period, bacteria and phages have co-evolved to outcompete one another. Bacteriophages and phage-derived proteins have shown promising results against MDR pathogens. In 2017 Dr Tom Patterson became the first person to be intravenously treated using a personalized ΦPC phage against multidrug-resistant *A.baumannii* systemic infection (Schooley et al., 2017). Since all the AMDs had failed, his doctors had decided to try one last luck using phages as he was in a near-death coma. Fortunately, the therapy worked, and Tom did not show any after effects post-treatment. However, there are many hurdles to be cleared before establishing successful phage therapy on a global scale. The strain specificity of phages hinders mass production, making the output quite labour-intensive and expensive (Loc-Carrillo and Abedon, 2011). The inability to replicate the *in-vitro* results in the actual situations still points to the lack of knowledge about the functioning of phages (Górski et al., 2020). Simultaneously the possibility of phages contributing to the AMR development through transduction still lies as a major concern (Principi et al., 2019).

### CRISPR-Cas9 Antimicrobials

CRISPR-cas9 system, even though regularly used as a tool for genetic modification in studies and a defence mechanism against phage attacks by bacteria, is recently being tested for being an addition to the artillery against AMR (Bikard et al., 2014)^,^ (Citorik et al., 2014). This stealthy approach works by introducing a spy to the population who would cripple the organization from the inside. Here the spy is none other than a bacteria or bacteriophage armed with the CRISPR cas9 system to target resistant genes. The study conducted was successful in *E.coli* and *S.aureus* (Bikard et al., 2014)^,^ (Citorik et al., 2014). However, the technique is far away from its maximum potential. First, the vast diversity of species within a microbiome and strains within a bacterial population makes targeting difficult (Pursey et al., 2018). There is a very high chance of causing an imbalance in the microbiome, resulting in the rise of a suppressed community of lethal pathogens that otherwise remain hidden. Next comes the central issue of gaining resistance against the CRISPR Cas9 by the positive selection of anti CRISPR (acr) gene (found in *Pseudomonas aeruginosa*) (Borges et al., 2017)^,^ (Pawluk et al., 2018). The delivery mechanism so far available is through conjugative plasmid harbouring bacteria and phages (Thomas and Nielsen, 2005). Still, the narrow host range, reduced uptake, and difficulty establishment are significant concerns when going against complex microbial communities.

### Drug Repurposing

All the above discussed approaches are time consuming and require a lot of financial resources involved in screening and standardization-based studies. The re-investigation of the previously known drug as a new treatment option for another disease use can help buy enough time for other techniques to be established or rise. This process is known as Drug Repurposing, which is a widely accepted phenomenon in the case of cancer and various other ailments (Zhang et al., 2020). Drug repurposing saves money and time as the drugs have already been through all the toxicology and human studies. A new medication that would cost at least an investment worth 1.3 Billion USD on an average with a minimum period of 10 years while repurposing costs less than 60% of this and 3–12 years (Rudrapal et al., 2020). Since 2014 drug repurposing of antimicrobials has gained momentum as ∼1900 reports have been published ever since. 2020 saw the highest surge in antimicrobial drug repurposing research publications.

The potential of drug repurposing against battling AMR has led to the rise of many joint ventures by various research organizations and pharmaceutical companies. Initiatives like Medicines of Malaria Venture (MMV), Drugs for Neglected Diseases Initiative (DNDi), Takeda, Daiichi Sankyo, Eisai, Calibr, and The Helmholtz Institute for Pharmaceutical Research Saarland (HIPS) provide researchers across the globe with drug screening libraries consisting of known drugs and potential drug molecules (MMV, 2021)^,^ (DNDi, 2021). This helps to screen at a far greater scale various samples and even helps find molecules against neglected diseases. Simultaneously establishing online drug databases has aided in the high throughput in-silico screening for potential drug targets and drug molecules further expediting the repurposing process. Drug repurposing and multidrug therapy have enough capacity to pack some deadly blows to AMR infections. However, like any other method, this approach involves some drawbacks like the reliability issues involving in-silico screening, pricing of drugs and patent related issues (Gill et al., 2016).

### Vaccination Against Pathogens

Vaccination is an efficient approach that over the years have helped humanity tackle various diseases and since the past few years the number of vaccines licensed are more in number than AMDs (Bloom et al., 2018). Vaccine administration reduces the chances of undergoing AMD treatment upon pathogen exposure, leading to a drop in the selection of AMR variants (Buchy et al., 2020). This reduction in selection pressure is an extraordinary feature that various other approaches fail to achieve. Recently many studies have shown that vaccination of farm animals are capable of minimizing the antibiotic footprint related to the food industry (Adam, 2009)^,^ (van Dommelen and Wertenbroek, 2011). There is a critical overuse of AMD in feeds of food animals to prevent animals from falling sick. It leads to heavy inflow of AMDs into the food chain resulting in AMR pathogen outbreak. The classic example is that of *Clostridium tetani,* despite of having high prevalence in the environment high infective potential, due to effective vaccination against it has helped reduce number of infection rates. At the same time the reports of drug resistant *C.tetani* is bare minimal, even though they harbour the potential to develop resistance against drugs upon continuous exposure (Rodrigo et al., 2014). This is an evidence that vaccination have the potential to deal heavy impact against rise of AMR, as it prepares the host body to fight the pathogen (Rosini et al., 2020) which indirectly reduces antibiotic administration. There has been successful implementation of vaccination against *Haemophilus influenza* (Peltola et al., 1999) and *Streptococcus pneumonia* (Jansen et al., 2018) which too, over the year have managed to reduce the infection rates and emergence of drug resistant variants of the respective pathogens Table 1.

**TABLE 1:** List of Non-conventional drugs under clinical trials or FDA approved against bacterial pathogens.

Sr.no	Antimicrobial	Type of antimicrobial	Status	Mode of action	Target pathogen	Reference
1	PLG0206 (WLBU2)	Antimicrobial Peptide	Phase-I	membrane permeability, remaining targets yet to be found	*P. aeruginosa, A. baumannii, Enterobacterales, S. aureus, S. pneumoniae*	[73]
2	SPR-206	Antimicrobial Peptide	Phase-I	lipopolysaccharide to disrupt the outer membrane	*P. aeruginosa, A. baumannii, Enterobacterales, S. aureus, S. pneumoniae*	[74]
3	Tryptophan-containing peptides	Antimicrobial Peptide/ Antivirulence Drug	Pre-clinical	Downregulation of quorum Sensing associalted virulence factors	*P. aeruginosa*	[75]
4	AR-101 (panobacumab, Aerumab)	Antivirulence Drug	Phase-IIa	O-antigen	*P. aeruginosa*	[76]
5	AR-301 (tosatoxumab)	Antivirulence Drug	Phase-III	alpha-toxin	*S. aureus*	[77]
6	CAL02	Antivirulence Drug	Phase-I	unknown mechanism, but toxins are inhibited	*P. aeruginosa, A. baumannii, Enterobacterales, S. aureus, S. pneumoniae*	[78]
7	AB103 (reltecimod)	Host Directed Therapy/ Antivirulence Drug	Phase-III	CD 28 T-lymphocyte receptor mimetic, inhibits T-cell stimulation	*S. aureus*	[79]
8	CC-11050	Host Directed Therapy	Phase-II	unknown	*M. tuberculosis*	[80]
9	Everolimus	Host Directed Therapy	Phase-II	downregulate lipid content within the foamy macrophages	*M. tuberculosis*	[81]
10	Imatinib	Host Directed Therapy/Repurposed Drug	FDA Approved	disrupts cellular mechanism used by Mtb for entry and survival in host cells	*M. tuberculosis*	[82]
11	Metformin	Host Directed Therapy/Repurposed Drug	FDA Approved	expands CD8+CXCR3+ TM cells in mice and humans, reprograms CD8^+^ T cells metabolic and transcriptional circuits	*M. tuberculosis*	[83]
12	Prednisone	Host Directed Therapy/Repurposed Drug	Phase-IV	unknown	*S. pneumoniae*	[84]
13	StaphVAX	Vaccine	Phase-III	capsular polysaccharides type 5 (CP5) and CP8	*S. aureus*	[85]
14	ExPEC4V	Vaccine	Phase-II	O antigens targeting all 4 serotypes	*E. coli*	[86]
15	M protein: 26-valent N-terminal	Vaccine	Phase-II	M Protein	*S. pyogenes*	[87]
16	LBP-EC01	Cas9 Antimicrobial/Phage Therapy	Phase-II	Natural lytic activity of the bacteriophage along with the DNA-targeting activity of CRISPR-Cas3	*E. coli, K. pneumoniae*	[88]
17	PhageBank	Phage Therapy	Phase-I/II	Bacteriophage mediated bacterial cell lysis	*E. coli, K. pneumoniae*	[89]
18	SER-109	Live Biotherapeutic	Phase-III	Purified Firmicutes spores	*C. difficle*	[90]
19	VE303	Live Biotherapeutic	Phase-II	8 clonal human commensal bacterial strains manufactured under GMP conditions	*C. difficle*	[91]
20	CP101	Live Biotherapeutic	Phase-II	Cocktail of commensal bacterial strains	*C. difficle*	[92]

However like any other method successful implementations of vaccination too have a lot of obstacles that needs be cleared. The first and foremost being the varying accessibility to vaccination on a global scale on the basis of health infrastructure and economy of the region. Accessibility will affect the global coverage of vaccination giving high birth rate countries like India and China the shorter end of the stick. Next is the lack of awareness about vaccine among various populations this makes the implementation of vaccine difficult. There was an increase of 3.2 million completely unvaccinated children globally in 2020 WHO report (WHO, 2020). The global coverage itself has dropped from 86% to 83% in the span from 2019 to 2020 (WHO, 2020). The development and implementation of a single vaccine involves approximately $1.2 billion–$8.4 billion and 10years or more of time (Gouglas et al., 2018)^,^ (Plotkin et al., 2017). This amount of money is not something all countries can afford to invest, as there are 64 countries with GDP less than even $10 billion (Author Anonymous, 2022). But with the involvement of bodies like WHO and UNICEF this economic and distribution based obstacles are being overcome. The proper focusing of developing vaccines against pathogens based on necessity and severity can help tackle the situation in a systematic manner.

### Live Biotherapeutic

Probiotics-based items have gained massive boost in sales over the past few years (Silva et al., 2020). Recognizing and understanding the importance of human microflora has led to this rise of probiotics usage as supplements. Probiotics are living microorganisms beneficial to the host, forming a symbiotic relationship between the two when ingested in an adequate amount (Pickard et al., 2017). The host microflora can assist in inhibiting the growth of pathogenic bacteria (Wolvers et al., 2010). When AMD therapy is given to the host, its microflora gets disrupted, and the drug-resistant variants of pathogenic bacteria get selected; at this point probiotic therapy comes to the rescue. The probiotics will help restore the microflora disrupted by the drug therapy lowering the chances of the resistant variant development. In the current times, the probiotic bacteria are being engaged in antimicrobial therapy as ‘Live Biotherapeutic’. The primary aims of Live Biotherapeutic therapy are; 1) Reduce the after-effects from traditional antimicrobial therapy, 2) reduce the required dosage of classical drugs, 3) faster healing of patients, 4) efficient and faster clearance of pathogens (Wolvers et al., 2010). The ability of these probiotic organisms to block the binding of pathogens to host receptors, production of antimicrobial substances, enhance host immune surveillance, and inflammatory responses will help achieve the goals mentioned above. Various studies have shown a synergistic effect against bacterial and fungal infections upon using conventional antimicrobial therapy with probiotics. *Helicobacter pylori* are one of the most difficult to eradicate bacterial pathogens. However, 10% improvement in eradication rate was observed when probiotics were administered and standard treatment (Caselli et al., 2016). Similarly, a study showed that use of probiotics-based sanitization in hospital helped reduce the frequency of AMR genes harbored by surface hospital microbiota at up to 99% (Montassier et al., 2021). The timing of probiotic treatment is very crucial, recently a study showed there was a drop in the number of AMR genes when given before AMD therapy (Tóth et al., 2021). However, AMR gene numbers remained unchanged when probiotic treatment was prescribed post AMD therapy (Tóth et al., 2021).

Many obstacles need to be overcome before using a Live biotherapeutic as treatment option. There is a big chance of AMR gene transfer from probiotic bacteria to other pathogens in the environment and vice versa (Zheng et al., 2017). Strict surveillance is required to avoid the usage of bacteria harboring transferable antimicrobial resistance genes. The proper guidelines for evaluating selected probiotic microorganisms capability and its dosage as an antimicrobial therapy are still lacking. Excessive usage of probiotics is reported to cause various complications over time (Bergman et al., 2020), hence, making dosage regulation a crucial aspect for successful implication.

### Host Directed Therapy

The host-directed therapy (HDT) aims to fight the bacterial infection by targeting host cell pathways modulated by the bacteria for its benefit. Since the treatment will be targeting host molecules, there will be no direct selection pressure on the pathogens. Hence such indirect targeting of pathogens can reduce the rise of drug-resistant variants to a greater extent. The bacterial pathogens have evolved multiple ways to evade the host immune system over the years. HDT majorly focuses on overcoming this evasion and continuing host cell pathways to eradicate pathogens. This pathogen clearance is achieved by triggering AMPs, reactive oxygen species (ROS and NO), and autophagy in host cells (Brighenti et al., 2018). *Mycobacterium tuberculosis (Mtb)*is a deadly pathogen that is difficult to treat due to its top-notch immune evasion mechanism and antimicrobial resistance profile. Mtb is phagocytosed by macrophages and inhibits the maturation of endosomes. Mtb inhibits the fusion of phagosome and lysosome and egress into the cytosol, where they recruit lipid bodies. Numerous HDT based approaches have shown promising results against Mtb, through steroid and non steroid based anti-inflammatory drug and Phosphodiesterase inhibitors by regulating the host inflammatory response (Baindara, 2019). Recondition the macrophage into the adequate clearance of Mtb is another aspect that’s of great interest to tackle Mtb based infection. Vitamin-D is highly effective against Mtb, as it enhances antimicrobial peptide production, induces autophagy, and inhibits lipid body formation (Delsing et al., 2014). Studies have shown, incredible results on HDT’s ability to subdue sepsis (Parihar et al., 2019)^,^ (Hotchkiss et al., 2013). Most death associated with sepsis occurs when it lasts for more than 3 days leading to an immunosuppressive state (Chang et al., 2014). Restoration of the host immune system during the immune-suppressive phase can help save the patient from death. Two of the most prominent HDT against sepsis are cytokine therapy during the immune-suppressive phase and blocking of Programmed Death (PD1) and Programmed Cell Death Ligand 1 (PD-L1) associated pathways (GDP, 2021).

The majority of the HDT drugs can be developed through repurposing, creating a faster route from the lab to market. However, authorities should make stringent guidelines to check their cytotoxicity and their chances of deteriorating patients conditions. Further, more profound studies about host-pathogen interaction between various pathogens can help develop HDT for different microbial infections.

## Outlook

We are currently at a crucial time; each action taken will determine the future of humanity in the battle against AMR. Many newer approaches for tackling this issue are evident with establishing bodies like Global Antibiotic Research and Development (GARDP) and CARB-X (GARDP, 2022)^,^ (Alm and Gallant, 2020). There is an excellent boost in developing the improved or newer approach. Delivery is a central lagging area in most of the approaches (Table 1). Improvement of drug delivery to the target shall help increase the efficiency and effectiveness of the treatment. The introduction of liposomes as a mode of AMD delivery in target cells is a new approach (Gonzalez Gomez and Hosseinidoust, 2020). The Healthcare system can further utilize CRISPR antimicrobial and phage DNA delivery. With the rising interest in AMPs, its conjugation with AMDs will aid in drug discovery and drug repurposing. However, for an overall victory against AMR, the selection pressure by the conventional AMDs needs to be abolished.

Newer drugs and techniques need to be developed that targets more non-essential aspects of the pathogen for its disarmament and rely more on the immune system to fight the battle. The host-pathogen interactions related to various infectious pathogens will help lay the foundation for perfecting indirect techniques to defeat pathogens. Conventional medicines should only be used in people with weak immunity or immunocompromised patients.

## Abbreviations

AMD, antimicrobial drugs; AMP, antimicrobial peptides; AMR, antimicrobial resistance; AVD, Antivirulent Drugs; CDC, Centre for Disease Control; CARB-X, combating antibiotic resistant bacteria; DNDi, drugs for neglected Diseases Initiative; FDA, food and drug Administration; GARDP, global antibiotic research and development; HIPS, The Helmholtz Institute for Pharmaceutical Research Saarland; MDR, Multidrug Resistance; MMV, medicines of malaria venture; MRSA, Methicillin Resistant *Staphylococcus aureus*; PDR, pan drug resistance; ROS, reactive oxygen species, VISA, vancomycin intermediate *Staphylococcus aureus*; WHO, world health organization; XDR, extended spectrum drug resistance.
